# Supplementary material for: Pyruvate Dehydrogenase Kinase 1 inhibition mediated oxidative phosphorylation enhancement in cartilage promotes osteoarthritis progression
Source: BMC Musculoskelet Disord. 2023 Jul 20;24:597. doi: 10.1186/s12891-023-06585-6 (PMC10357736; doi:10.1186/s12891-023-06585-6)
Supplement: Supplementary file 1 — Additional file 1. [file 12891_2023_6585_MOESM1_ESM.zip › MF.Gsea.1653627316242/GOMF_OXIDOREDUCTASE_ACTIVITY_ACTING_ON_A_SULFUR_GROUP_OF_DONORS.html]

Details for gene set GOMF\_OXIDOREDUCTASE\_ACTIVITY\_ACTING\_ON\_A\_SULFUR\_GROUP\_OF\_DONORS[GSEA]

|  || Dataset | 55457\_collapsed\_to\_symbols.cls.cls #OA\_versus\_normal.cls.cls #OA\_versus\_normal\_repos |
| Phenotype | cls.cls#OA\_versus\_normal\_repos |
| Upregulated in class | OA |
| GeneSet | GOMF\_OXIDOREDUCTASE\_ACTIVITY\_ACTING\_ON\_A\_SULFUR\_GROUP\_OF\_DONORS |
| Enrichment Score (ES) | 0.48054132 |
| Normalized Enrichment Score (NES) | 1.8895851 |
| Nominal p-value | 0.0 |
| FDR q-value | 0.021790335 |
| FWER p-Value | 0.295 |
Table: GSEA Results Summary

  

Fig 1: Enrichment plot: GOMF\_OXIDOREDUCTASE\_ACTIVITY\_ACTING\_ON\_A\_SULFUR\_GROUP\_OF\_DONORS      
 Profile of the Running ES Score & Positions of GeneSet Members on the Rank Ordered List

  

| SYMBOL | TITLE | RANK IN GENE LIST | RANK METRIC SCORE | RUNNING ES | CORE ENRICHMENT || 1 | GLRX5 | glutaredoxin 5 [Source:HGNC Symbol;Acc:HGNC:20134] | 263 | 0.778 | 0.0423 | Yes |
| 2 | PDIA5 | protein disulfide isomerase family A member 5 [Source:HGNC Symbol;Acc:HGNC:24811] | 324 | 0.735 | 0.0970 | Yes |
| 3 | MSRA | methionine sulfoxide reductase A [Source:HGNC Symbol;Acc:HGNC:7377] | 380 | 0.715 | 0.1504 | Yes |
| 4 | DNAJC10 | DnaJ heat shock protein family (Hsp40) member C10 [Source:HGNC Symbol;Acc:HGNC:24637] | 410 | 0.702 | 0.2048 | Yes |
| 5 | MSRB2 | methionine sulfoxide reductase B2 [Source:HGNC Symbol;Acc:HGNC:17061] | 412 | 0.702 | 0.2614 | Yes |
| 6 | ENOX1 | ecto-NOX disulfide-thiol exchanger 1 [Source:HGNC Symbol;Acc:HGNC:25474] | 558 | 0.638 | 0.3016 | Yes |
| 7 | DLD | dihydrolipoamide dehydrogenase [Source:HGNC Symbol;Acc:HGNC:2898] | 565 | 0.635 | 0.3523 | Yes |
| 8 | SELENOT | selenoprotein T [Source:HGNC Symbol;Acc:HGNC:18136] | 639 | 0.609 | 0.3958 | Yes |
| 9 | P4HB | prolyl 4-hydroxylase subunit beta [Source:HGNC Symbol;Acc:HGNC:8548] | 777 | 0.575 | 0.4315 | Yes |
| 10 | GFER | "growth factor, augmenter of liver regeneration [Source:HGNC Symbol;Acc:HGNC:4236]" | 1075 | 0.500 | 0.4488 | Yes |
| 11 | TXNRD3 | thioredoxin reductase 3 [Source:HGNC Symbol;Acc:HGNC:20667] | 2049 | 0.356 | 0.4019 | Yes |
| 12 | PDIA6 | protein disulfide isomerase family A member 6 [Source:HGNC Symbol;Acc:HGNC:30168] | 2073 | 0.353 | 0.4286 | Yes |
| 13 | PRDX3 | peroxiredoxin 3 [Source:HGNC Symbol;Acc:HGNC:9354] | 2161 | 0.343 | 0.4495 | Yes |
| 14 | SQOR | sulfide quinone oxidoreductase [Source:HGNC Symbol;Acc:HGNC:20390] | 2225 | 0.336 | 0.4717 | Yes |
| 15 | SELENBP1 | selenium binding protein 1 [Source:HGNC Symbol;Acc:HGNC:10719] | 2434 | 0.310 | 0.4805 | Yes |
| 16 | CCS | copper chaperone for superoxide dismutase [Source:HGNC Symbol;Acc:HGNC:1613] | 2727 | 0.278 | 0.4802 | No |
| 17 | GLRX3 | glutaredoxin 3 [Source:HGNC Symbol;Acc:HGNC:15987] | 3336 | 0.218 | 0.4505 | No |
| 18 | ERO1A | endoplasmic reticulum oxidoreductase 1 alpha [Source:HGNC Symbol;Acc:HGNC:13280] | 3686 | 0.187 | 0.4384 | No |
| 19 | PDIA4 | protein disulfide isomerase family A member 4 [Source:HGNC Symbol;Acc:HGNC:30167] | 3756 | 0.181 | 0.4477 | No |
| 20 | ENOX2 | ecto-NOX disulfide-thiol exchanger 2 [Source:HGNC Symbol;Acc:HGNC:2259] | 3769 | 0.179 | 0.4612 | No |
| 21 | SUOX | sulfite oxidase [Source:HGNC Symbol;Acc:HGNC:11460] | 3801 | 0.177 | 0.4731 | No |
| 22 | TXNL1 | thioredoxin like 1 [Source:HGNC Symbol;Acc:HGNC:12436] | 4135 | 0.143 | 0.4587 | No |
| 23 | TMX2 | thioredoxin related transmembrane protein 2 [Source:HGNC Symbol;Acc:HGNC:30739] | 4245 | 0.133 | 0.4610 | No |
| 24 | GSR | glutathione-disulfide reductase [Source:HGNC Symbol;Acc:HGNC:4623] | 4597 | 0.105 | 0.4421 | No |
| 25 | TMX1 | thioredoxin related transmembrane protein 1 [Source:HGNC Symbol;Acc:HGNC:15487] | 5101 | 0.064 | 0.4082 | No |
| 26 | IFI30 | IFI30 lysosomal thiol reductase [Source:HGNC Symbol;Acc:HGNC:5398] | 5483 | 0.033 | 0.3812 | No |
| 27 | MSRB1 | methionine sulfoxide reductase B1 [Source:HGNC Symbol;Acc:HGNC:14133] | 5834 | 0.006 | 0.3545 | No |
| 28 | STAB1 | stabilin 1 [Source:HGNC Symbol;Acc:HGNC:18628] | 5966 | -0.002 | 0.3445 | No |
| 29 | TXN2 | thioredoxin 2 [Source:HGNC Symbol;Acc:HGNC:17772] | 6072 | -0.010 | 0.3372 | No |
| 30 | GLRX2 | glutaredoxin 2 [Source:HGNC Symbol;Acc:HGNC:16065] | 6116 | -0.014 | 0.3349 | No |
| 31 | QSOX1 | quiescin sulfhydryl oxidase 1 [Source:HGNC Symbol;Acc:HGNC:9756] | 6130 | -0.015 | 0.3351 | No |
| 32 | GSTO1 | glutathione S-transferase omega 1 [Source:HGNC Symbol;Acc:HGNC:13312] | 6275 | -0.025 | 0.3260 | No |
| 33 | PCYOX1L | prenylcysteine oxidase 1 like [Source:HGNC Symbol;Acc:HGNC:28477] | 6330 | -0.031 | 0.3243 | No |
| 34 | PDIA3 | protein disulfide isomerase family A member 3 [Source:HGNC Symbol;Acc:HGNC:4606] | 6607 | -0.050 | 0.3068 | No |
| 35 | TXNRD1 | thioredoxin reductase 1 [Source:HGNC Symbol;Acc:HGNC:12437] | 6820 | -0.064 | 0.2955 | No |
| 36 | NXN | nucleoredoxin [Source:HGNC Symbol;Acc:HGNC:18008] | 7376 | -0.108 | 0.2611 | No |
| 37 | GLRX | glutaredoxin [Source:HGNC Symbol;Acc:HGNC:4330] | 7554 | -0.123 | 0.2573 | No |
| 38 | STAB2 | stabilin 2 [Source:HGNC Symbol;Acc:HGNC:18629] | 7680 | -0.133 | 0.2583 | No |
| 39 | ERO1B | endoplasmic reticulum oxidoreductase 1 beta [Source:HGNC Symbol;Acc:HGNC:14355] | 7954 | -0.152 | 0.2493 | No |
| 40 | PCYOX1 | prenylcysteine oxidase 1 [Source:HGNC Symbol;Acc:HGNC:20588] | 8235 | -0.174 | 0.2416 | No |
| 41 | PGK1 | phosphoglycerate kinase 1 [Source:HGNC Symbol;Acc:HGNC:8896] | 8513 | -0.195 | 0.2357 | No |
| 42 | PDIA2 | protein disulfide isomerase family A member 2 [Source:HGNC Symbol;Acc:HGNC:14180] | 8961 | -0.229 | 0.2194 | No |
| 43 | TXN | thioredoxin [Source:HGNC Symbol;Acc:HGNC:12435] | 9487 | -0.273 | 0.2006 | No |
| 44 | TXNRD2 | thioredoxin reductase 2 [Source:HGNC Symbol;Acc:HGNC:18155] | 9922 | -0.314 | 0.1922 | No |
| 45 | SCO2 | synthesis of cytochrome C oxidase 2 [Source:HGNC Symbol;Acc:HGNC:10604] | 11410 | -0.488 | 0.1159 | No |
Table: GSEA details [plain text format]

  

Fig 2: GOMF\_OXIDOREDUCTASE\_ACTIVITY\_ACTING\_ON\_A\_SULFUR\_GROUP\_OF\_DONORS      
 Blue-Pink O' Gram in the Space of the Analyzed GeneSet

  

Fig 3: GOMF\_OXIDOREDUCTASE\_ACTIVITY\_ACTING\_ON\_A\_SULFUR\_GROUP\_OF\_DONORS: Random ES distribution      
 Gene set null distribution of ES for **GOMF\_OXIDOREDUCTASE\_ACTIVITY\_ACTING\_ON\_A\_SULFUR\_GROUP\_OF\_DONORS**
